# The Dual Role of Gut Microbiota and Their Metabolites in Hepatocellular Carcinoma: A Context-Dependent Framework

**DOI:** 10.3390/microorganisms14010073

**Published:** 2025-12-29

**Authors:** Shuyu Zuo, Junhui Ma, Xue Li, Zhengyang Fan, Xiao Li, Yingen Luo, Lei Su

**Affiliations:** 1 NHC Key Laboratory of Human Disease Comparative Medicine, National Human Diseases Animal Model Resource Center, International Center for Technology and Innovation of Animal Model, Institute of Laboratory Animal Science, Chinese Academy of Medical Sciences (CAMS) & Comparative Medicine Center, Peking Union Medical College (PUMC), Beijing 100021, China; s2024007005@student.pumc.edu.cn (S.Z.);; 2 Cancer Hospital, Chinese Academy of Medical Sciences & Peking Union Medical College, Beijing 100021, China

**Keywords:** hepatocellular, carcinoma, gut microbiota, metabolites, context-dependency

## Abstract

Hepatocellular carcinoma (HCC) is a global health threat, and gut microbiota play a pivotal role in its pathogenesis through the gut–liver axis. However, the literature contains divergent or opposing findings: key microbial metabolites, such as secondary bile acids and indole derivatives, exhibit potent pro- and anti-tumorigenic activities across different studies, hindering a unified understanding of their veritable roles. To resolve this ambiguity, this review proposes a unifying “context dependency” framework. We posit that the functions of gut microbiota and their metabolites are not fixed but are dynamically determined by the host’s physiological and pathological “context,” defined here as the integrated dynamic background shaped by local metabolite concentrations, host immune status, specific receptor expression, and tumor microenvironment (TME) features. This framework is systematically substantiated through an analysis of the dichotomous effects of major microbial metabolites, including bile acids (BAs), short-chain fatty acids (SCFAs), trimethylamine N-oxide (TMAO), and indole derivatives. We further elucidate that the key “contextual factors” governing these functional outcomes include the TME, host immune status, metabolite concentration gradients, and the activation patterns of specific signaling pathways (e.g., farnesoid X receptor/takeda G protein-coupled receptor 5, aryl hydrocarbon receptor). This novel framework not only provides a theoretical foundation for integrating existing paradoxical findings but also paves the way for the future development of context-specific precision diagnostic biomarkers and targeted microbial intervention therapies for HCC.

## 1. The Relationship Between Gut Microbiota and Hepatocellular Carcinoma

Hepatocellular carcinoma (HCC) is the most common primary malignant tumor of the liver and is associated with high mortality [1]. Globally, there were 866,136 new cases and 758,725 deaths reported in 2022 [2]. Although diagnostic and therapeutic approaches have advanced, the majority of patients are diagnosed at intermediate or advanced stages due to complex genetic, epigenetic, and immune mechanisms [3], as well as the nonspecific nature of early symptoms [4], which significantly limits treatment efficacy and worsens patient prognosis. In both humans and animals, the gut microbiota exhibits remarkable species diversity [5,6,7,8,9]. With ongoing advances in intestinal microbiome research, numerous novel microbial species have been identified within this complex ecosystem [10,11,12,13,14,15,16,17,18,19]. Emerging evidence highlights the critical role these intestinal microorganisms play in maintaining host health and modulating disease pathogenesis [20]. Current studies increasingly reveal how microbial communities regulate diverse physiological processes, from nutrient metabolism to immune function, while dysbiosis has been linked to a range of disorders, including metabolic diseases, neurological conditions, and tumors [21,22,23,24,25].

A growing body of evidence indicates that the gut microbiota serves as a key regulator in the development and progression of various malignant tumors, earning it the title of “the microbial architect of malignant tumors”. In liver cancer, this role is particularly prominent through the gut–liver axis [26]. The interplay between gut microbiota and HCC has emerged as a major focus of research in recent years. As the body’s largest microbial ecosystem, the gut microbiota exerts dual regulatory effects through the bidirectional gut–liver axis, acting both as a guardian of hepatic homeostasis and a catalyst for pathological transformations [27]. Mounting evidence indicates that gut microbial communities contribute to HCC pathogenesis via multifaceted mechanisms, including metabolic reprogramming, immune dysregulation, and chronic inflammatory cascades [20,28]. A compromised intestinal barrier facilitates the systemic translocation of microbial components and bioactive metabolites, such as short-chain fatty acids (SCFAs), bile acids (BAs), and trimethylamine N-oxide (TMAO), which can promote hepatic inflammatory priming and fibrotic remodeling, thereby accelerating HCC progression [29,30]. Although current studies have identified several HCC-associated microbial signatures and their metabolic byproducts, key mechanistic questions remain unresolved. Notably, many microbiota-derived metabolites exhibit context-dependent duality, displaying both tumor-promoting and tumor-suppressive properties depending on microenvironmental conditions [20,28]. This functional duality underscores the need for deeper mechanistic investigation into host–microbe crosstalk during HCC development.

Dietary patterns critically shape the gut microbial ecosystem and its metabolic output, thereby influencing the gut–liver axis in HCC. Protective diets (e.g., high-fiber) promote beneficial bacteria and metabolites like SCFAs, while pro-carcinogenic diets (e.g., high-fat) can induce dysbiosis and increase translocation of harmful molecules (e.g., lipopolysaccharide) to the liver, fueling inflammation and carcinogenesis [31]. Thus, diet establishes a key “contextual layer” that modulates the gut microbiota’s role in HCC.

Despite the mounting evidence linking the gut microbiota to HCC, the field lacks a unified theoretical framework to account for the diversity and paradoxical nature of its effects. A simple dichotomous categorization through a simplistic beneficia versus detrimental dichotomy is scientifically insufficient and fails to capture the complexity of host–microbe interactions. Therefore, this review aims to fill this critical knowledge gap and introduce a “context-dependent” framework. Within this framework, “context” is defined as the dynamic pathophysiological background shaped by local metabolite concentrations, host immune status, specific receptor expression profiles, and tumor microenvironment characteristics. Herein, we will: (1) systematically review key microbial metabolites that exhibit dichotomous functional properties, using them as case studies to substantiate our core framework; (2) conduct an in-depth analysis of the molecular, cellular, and environmental contextual factors that act as functional switches for these metabolites; and (3) critically evaluate the potential and limitations of current microbial diagnostic and therapeutic strategies (e.g., fecal microbiota transplantation, engineered bacteria) through the lens of this new framework. Ultimately, we aim to provide researchers and clinicians with a more refined and dynamic model for understanding and targeting the gut microbiota in HCC.

## 2. Association Between Gut Microbiota and HCC

### 2.1. Landscape of HCC Microbiota

Gut dysbiosis, characterized by an imbalance in the microbial community, impairs antitumor immune surveillance and accelerates the progression from chronic liver disease to HCC [32]. This dysbiosis compromises intestinal barrier integrity, facilitating the translocation of bacterial components to the liver. Such translocation triggers systemic inflammation, which exacerbates hepatic injury and promotes HCC development [33]. As increased abundance of bacterial genera such as *Bacteroides* and *Clostridium* has been observed in patients with advanced HCC. These taxa modulate the tumor microenvironment, fostering conditions conducive to tumorigenesis [34].

### 2.2. Interaction Between Gut Microbiota and HCC

#### Gut–Liver Axis

**Figure 1:**
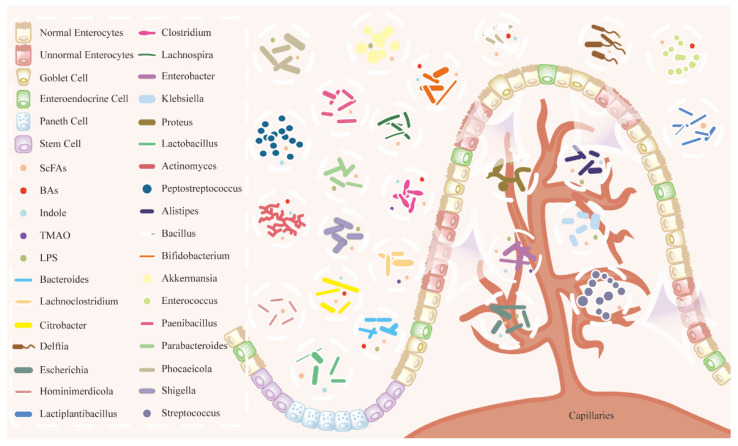
Translocation pathways of gut microbiota and metabolites across the intestinal barrier. This schematic illustrates key components of the gut–liver axis. Under physiological conditions, microbial metabolites (e.g., SCFAs can be selectively transported across the intact epithelium (represented by enterocytes and tight junctions) into the portal circulation, contributing to host metabolism and immune homeostasis. Under pathological conditions (e.g., barrier dysfunction represented by disrupted architecture), an increased translocation of microbes, their structural components (e.g., LPS), and other metabolites occurs, potentially driving systemic inflammation and liver disease. The figure lists representative bacterial genera and metabolites involved in these processes, highlighting the continuum from homeostasis to dysbiosis.

The gut–liver axis represents a bidirectional communication pathway through which gut microbiota-derived metabolites influence hepatic physiology, primarily via the portal circulation [28]. This axis plays a pivotal role in maintaining hepatic homeostasis and regulating metabolic functions. Dysregulation of fatty acid metabolism within this framework is strongly associated with metabolic dysfunction, inflammatory disorders, and the development of HCC [35]. Impaired intestinal barrier integrity facilitates the translocation of gut microbes and their metabolites to the liver through the portal vein [36]. Moreover, these microbial components and metabolites can enter systemic circulation via the lymphatic system, reaching the thoracic duct and subsequently disseminating throughout the body [37]. The gut microbiota and its metabolites are critical contributors to HCC pathogenesis. Specific bacterial species exhibit distinct roles, often displaying either pro-tumorigenic or anti-tumorigenic properties depending on contextual conditions. Both clinical and preclinical studies have established a robust association between microbial dysbiosis and HCC [38]. Evidence from animal and human research demonstrates a significant correlation between alterations in the gut microbiota and HCC progression (Table 1 and Table 2). This table framework has been optimized based on tables from reviews published in 2021 and supplemented with research data from 2021 onwards [35]. Shifts in microbial composition and metabolite profiles are now recognized as major factors driving HCC progression. The mechanism by which gut microbes enter into systemic circulation is illustrated in Figure 1.

### 2.3. HCC-Promoting Bacterial Species

Patients with HCC exhibit increased abundances of *Streptococcus*, *Shigella*, *Veillonella*, and *Acidaminococcus* compared to healthy controls. Reduced microbial diversity and specific compositional alterations, such as decreased *Blautia* and increased *Shigella*, compromise intestinal barrier integrity. This disruption promotes the translocation of bacterial metabolites (e.g., LPS) and viable bacteria (e.g., *Klebsiella pneumoniae*) to the liver, where they activate the Toll-like receptor 4 (TLR4)/nuclear factor kappa B (NF-κB) inflammatory pathway and thereby stimulate cancer cell proliferation [32,63,64]. Furthermore, dysbiosis contributes to HCC progression via immunosuppressive mechanisms, including the inhibition of CD8^+^ T cell function [30,32] and the downregulation of genes related to T cell receptor and natural killer (NK) cell activation, such as *CD6* and *MAPK10*) [34]. Additionally, microbial dysbiosis promotes angiogenesis and tissue necrosis through neutrophil extracellular traps (NETs), accelerating tumor dissemination [65]. It also exacerbates genomic instability via nucleotide-binding oligomerization domain-containing protein 2 (NOD2)-mediated nucleophagy and impaired DNA repair mechanisms [66]. Metabolic reprogramming, including aberrant BAs metabolism and reduced SCFAs levels, further worsens the hepatic microenvironment [34,64].

### 2.4. HCC-Protecting Bacterial Species

*Bifidobacterium*, *Coprococcus*, *Eubacterium*, and *Alistipes* may contribute to the prevention of HCC. The abundance of beneficial bacterial taxa, including *Bifidobacterium*, *Fusicatenibacter*, *Lachnospiraceae*, *Firmicutes*, and *Oscillospiraceae*, is significantly decreased in HCC patients compared to healthy controls [58]. These beneficial microbial communities play a crucial role in maintaining intestinal homeostasis (e.g., by enhancing intestinal barrier integrity) and in modulating immune responses (e.g., by promoting regulatory T cell differentiation and suppressing pro-inflammatory cytokine production). These effects are mediated primarily through the secretion of metabolites such as SCFAs and indole derivatives, which exert anti-tumorigenic effects [67]. Notably, specific probiotics and their metabolites demonstrate considerable potential for the prevention and treatment of HCC. For instance, *Bifidobacterium pseudolongum* produces acetate, an antitumor metabolite transported via the portal vein to the liver, where it directly inhibits the production of pro-inflammatory cytokines. Simultaneously, *B. pseudolongum* restores balance to the gut microbiome composition and enhances intestinal barrier integrity, further reducing systemic inflammation, and inhibit the progression of non-alcoholic fatty liver disease (NAFLD)-associated HCC [42]. Additionally, *Bifidobacterium longum* enhances tryptophan metabolism leading to the production of serotonin (5-HT), which facilitates hepatocyte proliferation and functional recovery [44]. Moreover, SCFAs derived from gut microbiota play a significant role in regulating anti-tumor immunity. For instance, acetate produced by *Lactobacillus reuteri* inhibits histone deacetylase (HDAC) activity, thereby reducing infiltration of interleukin-17A (IL-17A)-producing group 3 innate lymphoid cells (ILC3s) and improving the tumor immune microenvironment [43]. Additionally, it is important to distinguish between endogenous abundance and therapeutic potential. Dysbiosis in HCC is characterized by a general decrease in beneficial genera like *Bifidobacterium*, reflecting a loss of microbial diversity and homeostasis. However, exogenous administration of specific, functionally characterized probiotic strains can deliver a concentrated dose of protective metabolites (e.g., acetate) or directly modulate host immunity, thereby exerting therapeutic effects even in a dysbiotic background. However, further clinical validation is required for these probiotics to address challenges such as strain specificity and the need for personalized therapeutic strategies.

**Figure 2:**
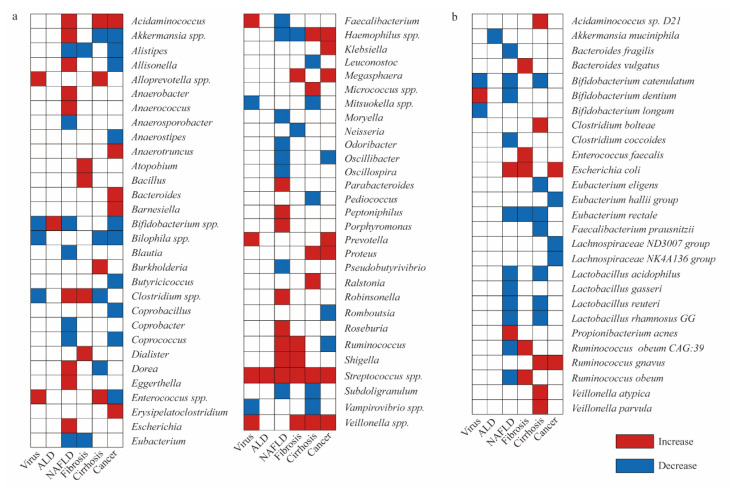
Compared to healthy individuals, patients with various liver diseases exhibit differences in the abundance of gut microbiota. (**a**) Changes in the abundance of gut microbiota at the genus level in patients with liver disease. *Streptococcus*, *Shigella*, *Veillonella*, and *Acidaminococcus* are associated with HCC development. *Bifidobacterium*, *Coprococcus*, *Eubacterium*, and *Alistipes* may help prevent HCC. (**b**) Changes in the abundance of gut microbiota at the species level in patients with liver disease. Virus: refers to hepatitis caused by viral infection; ALD: Alcohol-associated liver disease; NAFLD: Non-alcoholic fatty liver disease; red grid: increased microbial abundance compared to healthy individuals; blue grid: decreased microbial abundance compared to healthy individuals.

This study summarizes findings from several articles regarding changes in the abundance of gut microbiota in patients with liver disease. It provides a detailed overview of the specific microbial alterations associated with different types of liver conditions [68,69,70,71,72,73,74,75,76,77,78,79,80,81,82,83,84,85,86,87,88,89,90,91,92,93]. As summarized in this work, Figure 2 illustrates the changes in gut microbiota abundance in patients with various liver diseases compared to healthy controls.

## 3. Gut Microbiota Metabolites and Their Effects on HCC

**Figure 3:**
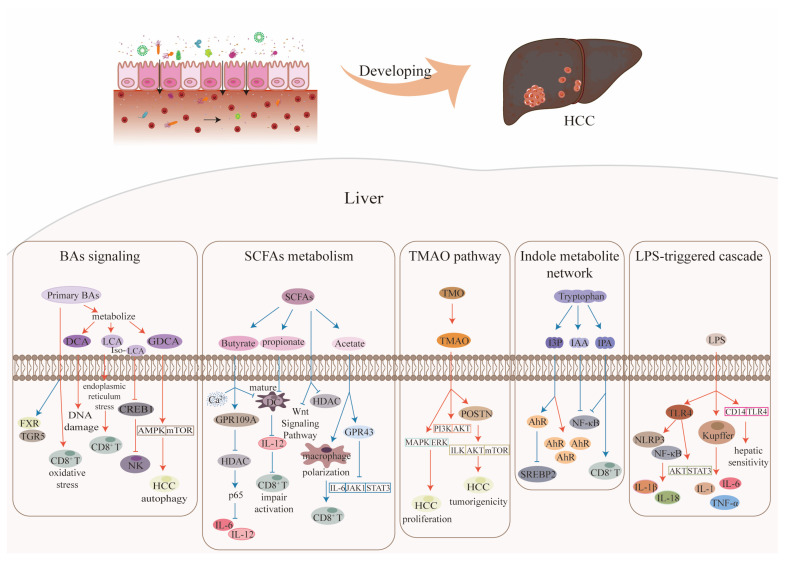
Mechanism diagram of gut microbiota-mediated pathogenesis in HCC via the gut–liver axis. The figure integrates key pathways through which gut microbial metabolites influence HCC development in a context-dependent manner. BAs signaling: Hydrophobic BAs (e.g., DCA) promote DNA damage, inflammasome activation, and suppression of anti-tumor immunity, while hydrophilic BAs (e.g., UDCA) exert protective effects via FXR/TGR5 activation and ER stress alleviation. SCFAs metabolism: SCFAs (butyrate, acetate, propionate) modulate HDAC activity, GPR43 signaling, and immune cell functions (e.g., ILC3s, CD8^+^ T cells), with outcomes depending on concentration, receptor expression, and microenvironmental pH. TMAO pathway: TMAO derived from gut microbial metabolism of choline promotes HCC progression via MAPK/ERK and ILK/AKT/mTOR axes, while its reduction attenuates fibrosis and inflammatory signaling. Indole metabolite network: Microbial tryptophan metabolites (e.g., IPA, I3P) differentially activate AhR, leading to either enhanced anti-tumor immunity (via T cell stemness) or immune tolerance and MYC-driven proliferation, depending on metabolite species and tumor-immune context. LPS-triggered cascade: LPS translocation activates TLR4-mediated NLRP3 inflammasome, mtDNA-cGAS-STING signaling, and hepatic stellate cell transdifferentiation, driving chronic inflammation, fibrosis, and tumor progression. In the diagram, the black arrow indicates the direction of movement, orange arrows indicate pro-carcinogenic pathways, while blue arrows denote tumor-suppressive pathways.

Gut microbiota participates in the body’s metabolism and produce various metabolites. This paper primarily summarizes the effects of these metabolites, bile acids (BAs), short-chain fatty acids (SCFAs), trimethylamine N-oxide (TMAO), indole metabolites, and lipopolysaccharide (LPS) on the development of liver cancer. The mechanistic contributions of the gut microbiome-gut–liver axis to HCC are summarized in Figure 3.

### 3.1. Bile Acids

BAs exhibit a profound functional dichotomy in HCC, serving as a quintessential paradigm of the “context-dependency” framework. The molecular basis for this duality is the vast chemical and signaling heterogeneity within the BAs pool, a feature dynamically shaped by the interplay between host hepatic synthesis and subsequent microbial biotransformation. The liver synthesizes primary BAs, which are relatively hydrophilic and function as high-affinity agonists for the farnesoid X receptor (FXR), a critical regulator of hepatic metabolic homeostasis [94]. A functional bifurcation is catalyzed by the gut microbiota, which metabolizes these precursors into structurally distinct and often more hydrophobic secondary BAs, such as deoxycholic acid (DCA). This enzymatic conversion precipitates a fundamental shift in signaling capacity: away from FXR agonism towards potent activation of the G-protein coupled receptor takeda G protein-coupled receptor 5 (TGR5), a key mediator of acute inflammatory and proliferative signaling [95].

The context-dependent nature of BAs manifests across two interrelated levels: First, the function of individual bile acid molecules can be reversed by changes in their concentration or alterations in the host microenvironment. For instance, while DCA at physiological concentrations contributes to normal epithelial cell turnover, pathologically elevated DCA levels in settings like non-alcoholic steatohepatitis (NASH) transform it into a potent “tumor catalyst” that accelerates HCC progression by inducing DNA damage and activating inflammasomes [96,97]. Even hydrophilic ursodeoxycholic acid (UDCA), which possesses protective effects, may see its efficacy diminished in certain late-stage liver diseases characterized by low FXR expression [96]. Second, the inherent physicochemical properties of different bile acids, such as DCA’s hydrophobicity versus UDCA’s hydrophilicity, constitute primary “contextual factors” determining their dominant biological characteristics (e.g., UDCA partially inhibits tumor growth by alleviating endoplasmic reticulum stress [98]). This structural duality, combined with dynamic regulation of abundance and signaling environments, collectively dictates final outcomes. Moreover, contextual factors extend to the systemic level, abnormal activation of the bile acid synthesis pathway impedes tumor-specific T cell responses, thereby promoting HCC progression [99]. This “double-edged sword” effect is not stochastic but is determined by the dynamic interplay among the host, microbiota, and metabolites [100].

High concentrations of hydrophobic BAs, such as DCA and chenodeoxycholic acid (CDCA), are recognized HCC “accelerators.” Their pro-tumorigenic mechanisms are multifaceted. Firstly, DCA, similar to other hydrophobic BAs like CDCA, can induce excessive mitochondrial reactive oxygen species (ROS) production and subsequent mitochondrial DNA (mtDNA) leakage, which directly activates the NLR family pyrin domain containing 3 (NLRP3) inflammasome, leading to the release of interleukin-1β (IL-1β) and interleukin-18 (IL-18) and ultimately driving hepatocyte pyroptosis and malignant transformation [97]. Secondly, DCA can inhibit FXR activity, its role in relation to FXR exhibits both weak agonistic and antagonistic properties, with antagonism being dominant in the HCC context. This inhibition relieves the restriction on the proliferation of Lgr5^+^ cancer stem cells, thereby promoting their expansion [96,101]. Lastly, as a secondary BA, DCA can also inhibit the expression of CXCL16 in liver sinusoidal endothelial cells, reducing the recruitment of natural killer T (NKT) cells, impairing hepatic anti-tumor immunity, and establishing an immunosuppressive microenvironment conducive to tumor escape [50]. These mechanisms are clinically substantiated, as elevated serum DCA levels in HCC patients correlate with tumor size and metastasis, and FXR-knockout mice spontaneously develop HCC in conjunction with an expanded DCA pool [96,102]. Additionally, isolithocholic acid (Iso-LCA) is a secondary bile acid produced by *Bacteroides ovale* metabolizing CDCA following *AKR1D1* deficiency. It inhibits NK cell secretion of interferon-γ (IFN-γ) and tumor necrosis factor-α (TNF-α) by reducing cAMP Response Element-Binding Protein 1 (CREB1) levels, thereby impairing NK cell cytotoxicity and ultimately promoting HCC progression [98].

In contrast, physiological concentrations or exogenous supplementation of hydrophilic BAs, such as UDCA, tauroursodeoxycholic acid (TUDCA), play the role of “extinguishing the flames” of carcinogenesis. UDCA effectively alleviates ER stress and inhibits oxidative stress in CD8^+^ T cells, thereby restoring sensitivity to anti-PD-1 (PD-1: programmed cell death protein 1) immunotherapy [99]. These protective effects of hydrophilic BAs hold significant translational potential. For instance, in murine models, genetic deletion of BAAT, a key enzyme in BA synthesis, reduces the production of harmful BAs and significantly lowers tumor burden; based on its role in protecting T cell function, it is speculated to indirectly enhance anti-PD-1 efficacy (not yet directly verified) [99]. Clinically, long-term administration of UDCA has been shown to reduce the risk of HCC in patients with primary biliary cholangitis (PBC) [102].

The functional balance of BAs is not static but is dynamically regulated by at least four key contextual knobs. The first is the molecular structure context, where the hydrophilicity and hydrophobicity of BAs form the basis of their function. DCA, with a hydrophobicity index greater than 0.5 [103], exhibits membrane-disrupting and genotoxic properties, whereas UDCA, with an index less than 0, acts as a membrane stabilizer and signaling molecule [104]. The second is the concentration-time context, with physiological concentrations of BAs acting as homeostatic ligands for FXR/TGR5 [105,106], while pathological high concentrations exhibit cytotoxicity [107]. The third is the host receptor expression context. In hepatocytes, functional FXR signaling suppresses pro-tumorigenic pathways like YAP/β-catenin [101,102]; however, in the context of low FXR expression, as seen in NASH, the protective signaling is lost, leading to BA metabolic disorders and activation of detrimental pathways [108]. The final knob is the tumor microenvironment context. In an acidic tumor microenvironment (TME), DCA can accelerate telomerase activity [107], whereas under high-fiber diet and probiotic interventions, the levels of UDCA increase, restoring NKT and NK cell immune surveillance [98,100].

This context-dependent perspective offers a rationale for clinical translation. For diagnostics, moving beyond single metabolites towards multi-metabolite signatures that reflect the overall metabolic context holds great promise. For instance, a prognostic model incorporating BAs profiles and gut microbiota signatures has been shown to effectively stratify HCC patient survival [50,109,110]. For therapeutics, novel strategies such as targeted UDCA delivery systems designed to release their payload preferentially in tumor compartments with low FXR expression could enable precision targeting (the protective role of UDCA in HCC is supported by previous studies [102,111,112], and targeted delivery technologies have been explored in liver cancer). For prevention, a strategy combining a high-fiber diet (which modulates BA metabolism and gut microbiota balance) with FXR agonists (e.g., obeticholic acid) is a rational approach to reduce the rate of NASH-to-HCC progression, given their established roles in metabolic regulation and alleviation of NASH-related liver injury [108]. The context-dependent dual roles of bile acids in HCC pathogenesis, mediated through structural specificity, receptor signaling (FXR/TGR5), and immunomodulatory effects, are visually synthesized within the metabolic network of the gut–liver axis in Figure 3.

### 3.2. Short-Chain Fatty Acids

SCFAs, primarily composed of acetic acid (C_2_), propionic acid (C_3_), and butyric acid (C_4_), these are saturated carboxylic acids. The length of their carbon chains influences their efficacy as histone deacetylase inhibitors and G protein-coupled receptor ligands. SCFAs exhibit a striking dichotomous function in HCC that is fundamentally context-dependent. Under physiological conditions, they act as potent inhibitors of tumor progression; however, this protective effect is lost or even reversed when the context is dysregulated, such as in cases of insufficient concentration or receptor absence. This functional paradox is ultimately dictated by a confluence of factors, including the host microenvironment, local SCFAs concentrations, the cellular receptor expression profile, and the specific SCFA-producing bacterial strains [113].

The protective effects of SCFAs are mediated through three core mechanisms. Firstly, butyrate, acting as a classic HDAC inhibitor at concentrations of 0.5–2 mmol/L, binds to the catalytic pocket of HDAC1/3, increasing the acetylation of *p65* to block the recruitment of NF-κB to the promoters of pro-inflammatory genes like interleukin-6 (IL-6) [114]. This epigenetic regulatory mechanism was robustly validated in the HBx transgenic mouse model, where oral administration of butyrate significantly reduced the incidence of HCC [115]. Secondly, acetate can bind to the G protein-coupled receptor 43 (GPR43) on hepatocytes, effectively blocking the IL-6/STAT3 (STAT3: signal transducer and activator of transcription 3) signaling pathway and leading to a significant reduction in JAK1 and STAT3 phosphorylation, respectively [42]. In a NAFLD-associated HCC mouse model, elevating portal vein acetate concentrations by gavage with *Bifidobacterium pseudolongum* resulted in a 67% reduction in tumor volume [42]. Furthermore, acetate produced by specific *Lactobacillus reuteri* can negatively regulate ILC3-IL-17A axis, significantly reducing IL-17A production by hepatic ILC3s while increasing the infiltration of CD8^+^ T cells into the tumor by 190% [43].

However, the protective effects of SCFAs are highly conditional and can be rapidly abrogated under dysregulated contexts. The most critical factor is the concentration threshold. When butyrate concentrations fall below physiological micromolar levels (approximately 100 μM), its function shifts significantly from HDAC inhibition to acting as an acetyl-CoA donor for *p300*. This may instead activate gene expression by promoting histone hyperacetylation [116]. These threshold-driven, non-linear concentration effects highlight that defining the precise “concentration window” is more critical than measuring absolute levels for biomarker design. Secondly, disruption of key receptor signaling, such as that of GPR43, also leads to a loss of protection. Indeed, GPR43-deficient mice fed a high-fat diet exhibit exacerbated obesity and insulin resistance, key risk factors that promote the progression towards NASH and HCC [117]. Moreover, the acidic tumor microenvironment (pH ≈ 6.5) can impair the function of SCFAs, as the protonated form of butyrate is less efficient in cellular uptake and HDAC inhibition, a principle that may limit its efficacy within the tumor core [118].

In summary, the functional balance of SCFAs is governed by at least four “contextual knobs”: SCFAs concentrations exhibit function-dependent effects: high concentrations within the colonic lumen (e.g., 0.5–5 mmol/L, the physiologically active range) primarily exert inhibitory effects, whereas low concentrations may activate *p300* [113,116]; the receptor expression profile, where high GPR43 expression mediates the anti-tumor effects of acetate, whereas low expression leads to signal loss [42]; the strain-specificity of production highlights that the functional outcome of a metabolite depends on its broader metabolic and immunological context: acetate derived from *Lactobacillus* species acts within an anti-inflammatory milieu, thereby inhibiting IL-17A, whereas acetate from *Bacteroides thetaiotaomicron* is accompanied by pro-inflammatory signals that promote M1 macrophage polarization [119,120]; and the TME pH/oxygen status, which can modulate SCFAs bioavailability and function [118]. This refined understanding informs clinical translation. For instance, developing diagnostic models that combine portal vein SCFAs fingerprints with GPR43 mRNA levels represents a promising, albeit yet to be validated, strategy for improving early HCC detection [42,43]. For therapeutics, novel approaches like pH-responsive butyrate nanoparticles designed to overcome the acidic TME barrier are an exciting area of future research. And for prevention, combining prebiotics like inulin with specific probiotics offers an evidence-based strategy for preventing NAFLD-to-HCC progression [42,115,121]. The concentration- and receptor-dependent mechanisms by which SCFAs exert pro- or anti-tumorigenic effects in HCC are integrated into the overarching pathway map presented in Figure 3.

### 3.3. Trimethylamine N-Oxide

TMAO is a zwitterionic small molecule with the structure (CH_3_)_3_N^+^-O^−^, formed by the oxidation of trimethylamine produced by gut microbiota in the liver. The function of TMAO in HCC is not singularly detrimental but rather exhibits significant context-dependency. In the pathological context of chronic high concentrations, it acts as a promoter of inflammation and tumor progression; however, at physiological concentrations or under specific interventions, it can alleviate fibrosis and exert compensatory protective effects. This functional duality is co-determined by plasma concentration, the abundance of gut microbial trimethylamine (TMA) producers, the activity of hepatic flavin-containing monooxygenase 3 (FMO3), and the host’s overall metabolic state [122,123].

The pro-tumorigenic effects of TMAO are mediated through at least three core mechanisms. Firstly, at concentrations exceeding 50 μmol/L, TMAO can activate the MAPK pathway, upregulating the level of p-ERK in HCC cells and thereby promoting the G1/S phase transition of the cell cycle [124]. Secondly, TMAO can induce the secretion of periostin (POSTN), which in turn activates the integrin-linked kinase (ILK)-AKT-mTOR signaling axis, leading to epithelial–mesenchymal transition (EMT) in HCC cells [125]. In animal models, TMAO treatment increased subcutaneous tumor volume by 2.4-fold, an effect that was completely abolished by POSTN knockdown [125]. These mechanisms are strongly supported by clinical evidence; a case–control study in China demonstrated a dose-dependent positive association between plasma TMAO levels and the risk of primary liver cancer, with a multivariable-adjusted OR of 2.85 for the highest vs. lowest quartile [60].

However, under specific contextual shifts, the function of TMAO can be dramatically reversed. For instance, in a cholestatic liver fibrosis model, intervention with traditional Chinese medicine that inhibited intestinal TMA-producing bacteria (such as *Prevotella copri*) led to decreased plasma TMAO levels and, paradoxically, alleviated PI3K/AKT pathway-mediated liver fibrosis [126]. Furthermore, at physiological concentrations, TMAO can act as an osmolyte and chemical chaperone, stabilizing protein folding and thus mitigating endoplasmic reticulum stress [123]. Intriguingly, in certain human populations, TMAO levels are inversely correlated with disease severity. For example, a study on obese individuals with type 2 diabetes found that subjects with higher plasma TMAO levels had a lower incidence of NASH [127].

In summary, the functional balance of TMAO is governed by at least four “contextual knobs”: the plasma concentration threshold, with physiologically low concentrations exerting protective effects while physiologically high concentrations promotes proliferation [123,124]; the abundance of gut TMA-producing bacteria, where a high abundance of species like *Prevotella copri* is a prerequisite for elevated cancer risk [128]; the hepatic FMO3 expression level, where high FMO3 expression amplifies the pro-tumorigenic signal, but its absence can induce liver damage due to TMA accumulation [122]; and the host metabolic background, where TMAO may play a compensatory protective role in early-stage NASH but drives malignant transformation in late-stage carcinogenesis [127]. This refined understanding opens new avenues for clinical translation. For example, combining plasma TMAO with POSTN can serve as a diagnostic biomarker to distinguish between cirrhosis and HCC [60,125], while targeting *Prevotella copri* or adopting a low-choline diet combined with FMO3 inhibitors offers promising strategies for the treatment and prevention of HCC [126,128]. The dichotomous function of TMAO in promoting inflammation or exerting compensatory protection, governed by microbial abundance and host metabolic state, is contextualized within the multi-metabolite interplay illustrated in Figure 3.

### 3.4. Indole Metabolites

Indole metabolites use tryptophan as a substrate; they share the indole ring (C_8_H_7_N) core structure, with differing side chains determining their functional variations (e.g., indole-3-propionic acid carries a propanoic acid side chain, indole-3-pyruvic acid carries a pyruvate side chain). Metabolites derived from the microbial processing of dietary tryptophan, such as indole-3-propionic acid (IPA), indole-3-acetic acid (IAA), and indole-3-pyruvic acid (I3P), exhibit a highly context-dependent function in HCC. Their roles are not simply beneficial or detrimental but are defined by a clear functional paradox: transient or low-level activation of the aryl hydrocarbon receptor (AhR) can promote anti-tumor immunity, whereas sustained high-level activation or the presence of a specific immune microenvironment induces immune tolerance and drives oncogene expression. This functional duality is co-determined by the specific metabolite species, the concentration and duration of exposure, the ligand affinity for AhR, and the immune status of the tumor microenvironment [129,130,131].

On one hand, within a protective context, indole metabolites inhibit HCC progression through multiple pathways. Firstly, IAA can effectively suppress lipogenesis and oxidative stress by downregulating hepatic fatty acid synthase and reducing malondialdehyde levels [132]. Secondly, IPA plays a critical role in maintaining the integrity of the gut–liver barrier; by upregulating the expression of tight junction proteins, it can reduce endotoxin levels in the portal vein by 67% [133]. Most importantly, IPA demonstrates significant potential in enhancing anti-tumor immunity. When combined with anti-PD-1 therapy, it can increase the proportion of stem-like CD8^+^ T cells (TCF1^+^) within the tumor by 2.8-fold, thereby substantially boosting immunotherapy efficacy and achieving a complete remission rate of 40% [134].

However, the function of these metabolites is reversed in dysregulated contexts, particularly within a tumor microenvironment (TME) rich in tumor-associated macrophages (TAMs). In this pathological milieu, the TAM-expressed enzyme IL4I1 preferentially converts tryptophan into I3P, a high-affinity AhR agonist [135,136]. This leads to a fundamental rewiring of AhR-mediated transcriptional responses. Unlike the transient AhR activation by low-affinity ligands from diet or commensal microbes that maintain immune homeostasis under physiological conditions [137], the sustained availability of I3P in the TME causes perpetual AhR signaling. This intense activation drives a distinct, pro-tumorigenic transcriptional program with dual effects: it upregulates PD-1 expression on CD8^+^ T cells [136], thereby inducing immune exhaustion, and it directly enhances the protein stability of the MYC oncogene within HCC cells to fuel their proliferation [138]. The clinical relevance of this mechanism is profound, as elevated I3P levels in the portal vein serum are strongly associated with poor prognosis in HCC patients; the three-year disease-free survival rate for patients with high I3P levels was only 26%, compared to 61% for the low-level group [139].

In summary, the functional balance of indole metabolites is governed by at least four “contextual knobs”: metabolite species and receptor affinity, where the weak agonist IPA causes transient activation while the potent agonist I3P leads to sustained activation [126,136,140]; the concentration-time window, where transient low concentrations promote T cell stemness while sustained high concentrations induce apoptosis [134]; the producing strains and metabolic pathways, such as *Bifidobacterium* species preferentially producing protective indole-3-lactic acid while Clostridium species favor the production of pro-tumorigenic I3P [140]; and the immune cell composition of the TME, where the presence or absence of TAMs is a key determinant of immune activation versus suppression [136]. For instance, the ratio of IPA to I3P in the portal vein emerges as a promising biomarker candidate for predicting anti-PD-1 response, a hypothesis built upon their opposing roles in T cell function and patient prognosis, respectively [134,139], designing engineered bacteria that specifically produce protective metabolites [140], and modulating microbial metabolism through a high-fiber diet have all shown immense translational promise [133,141]. The AhR-mediated duality of indole metabolites in modulating anti-tumor immunity or fostering immune tolerance is schematically represented within the tumor microenvironment network shown in Figure 3.

### 3.5. Lipopolysaccharide

LPS is a major component of the outer membrane in Gram-negative bacteria, a complex amphiphilic glycolipid who’s hydrophobic lipoteichoic acid domain is the key region for TLR4 activation. The biological effects of LPS in HCC are not uniformly detrimental but exhibit clear context-dependency. When the intestinal barrier is intact, trace amounts of LPS can act as a physiological immune adjuvant, maintaining the vigilance of the immune system. However, once the barrier is compromised, the continuous leakage of high-concentration LPS transforms it into a potent engine driving chronic inflammation and carcinogenesis. This functional duality is co-determined by the LPS concentration in the portal vein, the immune tolerance status of hepatic Kupffer cells (KCs), the efficiency of cellular mitophagy, and the pathological stage of liver fibrosis [142,143].

The pro-tumorigenic effects of LPS are primarily mediated through three core mechanisms. Firstly, upon binding to Toll-like receptor 4 (TLR4) on the surface of KCs, LPS rapidly recruits and activates the NLRP3 inflammasome, which cleaves Caspase-1 to release large quantities of IL-1β and IL-18, thereby creating a sustained pro-inflammatory microenvironment [144]. Secondly, LPS can induce DRP1-dependent mitochondrial fission, leading to the leakage of mitochondrial DNA (mtDNA) into the cytoplasm, which is then recognized by cyclic GMP-AMP synthase (cGAS) to activate the STING signaling pathway, ultimately resulting in a type I interferon storm that exacerbates liver injury [145]. Furthermore, LPS can also promote the transformation of hepatic stellate cells into myofibroblasts by upregulating *sphingosine kinase 1* in liver sinusoidal endothelial cells, which promotes the generation of sphingosine-1-phosphate (S1P), a potent positive feedback signal that accelerates the fibrotic process [146].

However, under specific contextual shifts, the function of LPS can be dramatically reversed or modulated. While high-dose LPS is overtly pro-inflammatory, the concept of “endotoxin tolerance”, where prior exposure to low-dose LPS desensitizes macrophages to subsequent challenges, is a well-established immunological principle [147,148,149]. This phenomenon significantly suppresses the secretion of pro-inflammatory cytokines like TNF-α. Concurrently, intrinsic cellular protective mechanisms, such as *ATG2B*-mediated mitophagy, can efficiently clear the cytoplasm of LPS-induced mtDNA, thereby blocking the excessive activation of the downstream STING signal [150]. Intriguingly, in a clinical setting, physiologically low levels of LPS are even positively correlated with faster postoperative liver regeneration [142], which may reflect its physiological role as a trace immune adjuvant.

In summary, the functional balance of LPS is governed by at least four “contextual knobs”: the portal vein concentration threshold, where physiologically low concentrations may be associated with regeneration while physiologically high concentrations trigger an inflammatory storm [145,151]; the tolerance status of KCs, where initial exposure triggers inflammation while repeated micro-dosing can induce tolerance [143]; the efficiency of cellular mitophagy, where efficient autophagy clears inflammatory signal sources and blocks the STING pathway [152]; and the stage of liver fibrosis, where the liver’s response to LPS is altered, with advanced fibrosis creating a vicious cycle of inflammation [146]. This refined understanding provides new avenues for clinical translation. For instance, developing diagnostic models that combine portal vein LPS with circulating mtDNA copy number represents a promising, albeit yet to be validated, strategy for predicting postoperative HCC recurrence [145,152]. For therapeutics, exosome-mediated delivery of *ATG2B* represents a potential strategy for mitigating liver injury [150]. Finally, maintaining intestinal barrier integrity and reducing portal LPS levels through prebiotic or other dietary interventions remains an effective strategy for preventing liver disease progression [151]. The concentration- and tolerance-dependent roles of LPS in driving inflammation or maintaining physiological immune vigilance are captured within the integrated gut–liver axis model summarized in Figure 3.

### 3.6. Role of Diet in Gut Microbiota–Host Interactions

Dietary patterns can exert bidirectional regulatory effects on the development of HCC by modulating the gut microbiota and its metabolites. Pro-carcinogenic diets, such as high-fat diets, can induce gut dysbiosis, increasing the abundance of pro-inflammatory bacteria (e.g., Gram-negative bacteria) while reducing beneficial bacteria (e.g., *Lactobacillus*, *Bifidobacterium*), thereby triggering leaky gut syndrome and facilitating the translocation of harmful metabolites, such as LPS and secondary BAs, into the liver [153]. High-cholesterol diets may disrupt the gut microbiota balance by elevating *Mucispirillum* and *Desulfovibrio* while depleting *Bifidobacterium* [39]. High-fructose diets can increase the abundance of acetate-producing bacteria, such as *Clostridia*, with microbial-derived acetate upregulating glutamine and UDP-GlcNAc levels, thereby enhancing protein O-GlcNAcylation in HCC and promoting tumor progression [154]. Conversely, protective diets, such as the Mediterranean diet or fiber-rich regimens, boost the abundance of SCFA-producing bacteria (e.g., *Akkermansia*, *Lactobacillus*). SCFAs inhibit inflammatory pathways via the GPR43 receptor, enhance CD8^+^ T cell-mediated antitumor immunity, and improve gut barrier function to reduce LPS translocation, thereby lowering the risk of non-alcoholic fatty liver disease (NAFLD) and HCC [155,156,157]. These findings highlight the pivotal role of the diet-gut microbiota-metabolism axis in HCC pathogenesis, providing a theoretical basis for dietary interventions targeting the gut microbiota to prevent or treat HCC.

### 3.7. Beyond Single Molecules: The Synergistic and Antagonistic Network of Microbial Metabolites

Microbial metabolites do not act in isolation but form a dynamic and interactive network of synergy and antagonism within the HCC microenvironment. This network utilizes the gut–liver axis as its main conduit for transport and the interplay among Kupffer cells, hepatocytes, and hepatic stellate cells as its central hub for signal processing. Its overall functional propensity, be it protective or pro-tumorigenic, is intricately regulated by four key “regulatory switches”: dietary patterns, intestinal barrier integrity, macrophage polarization status, and the concentration of key metabolites.

Protective metabolites likely act in synergy. For instance, both SCFAs like butyrate and indole metabolites like IPA are known to independently enhance intestinal barrier integrity by upregulating tight junction proteins, collectively leading to a significant reduction in LPS translocation from the gut to the portal vein [42,133]. Furthermore, potential crosstalk between SCFAs and BAs signaling pathways warrants investigation. It is plausible that SCFAs like acetate, via receptors such as GPR43, could modulate the activity of key nuclear receptors like FXR, potentially sensitizing them to protective bile acids like UDCA and thereby co-suppressing pro-tumorigenic pathways [42,99]. On an immunological level, multiple protective metabolites converge to enhance anti-tumor immunity. For instance, IPA enhances the efficacy of immunotherapy by modulating the stemness of CD8^+^ T cells [134]. The potential synergistic effects of these distinct pathways represent an exciting avenue for future combination therapies.

However, LPS and hydrophobic bile acids such as DCA act as a notorious “pro-tumorigenic duo.” Both metabolites are known to signal through TLR4 on KCs to promote inflammatory responses. It is therefore highly plausible that they act synergistically to amplify liver injury, for instance, by potentiating the activation of pathways like the NLRP3 inflammasome or the STING signaling cascade [96,145]. Similarly, synergistic pro-tumorigenic effects may arise from the interplay between TMAO and LPS. TMAO is known to promote HCC progression by inducing the secretion of POSTN [125], while persistent TLR4 activation by LPS also drives tumor growth [158]. The convergence of these two pathways could plausibly amplify HCC cell migration and invasion. Furthermore, LPS and I3P can form a malignant positive feedback loop: LPS stimulates TAMs to secrete the IL4I1 enzyme, leading to increased I3P production, which in turn exacerbates CD8^+^ T cell exhaustion through sustained AhR activation [136].

For instance, butyrate can directly antagonize the pro-inflammatory signal of LPS. Mechanistically, butyrate acts as an HDAC3 inhibitor, increasing the acetylation of the NF-κB subunit p65, which in turn prevents its binding to the promoters of pro-inflammatory genes and significantly suppresses the inflammatory response in macrophages [114]. Meanwhile, the hydrophilic UDCA, by activating the intestinal FXR-FGF19 axis, can significantly downregulate the hepatic transcription of FMO3, the key enzyme that oxidizes TMA to TMAO, thereby inhibiting TMAO generation at its source [108].

**Figure 4:**
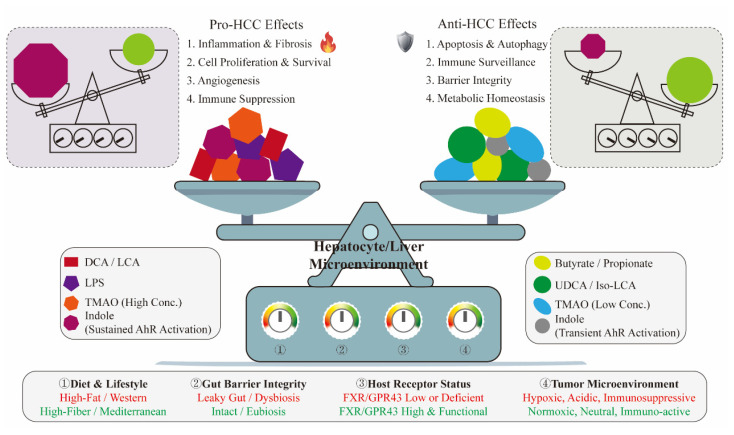
The Context-Dependent Functional Balance of Gut Microbial Metabolites in HCC. The flame symbol indicates a negative effect, while the shield symbol indicates a positive effect.

For diagnostics, integrated metabolomic analysis of portal vein serum revealed elevated levels of pathogenic metabolites (including DL-3-phenylglycolic acid, L-tryptophan, glycocholic acid, and 1-methylnicotinamide) were elevated, while protective metabolites (linoleic acid and phenolic compounds) were reduced. A partial least squares discriminant analysis (PLS-DA) model effectively distinguished HCC patients from healthy individuals [139]. For therapeutics, novel strategies such as pH-responsive nano co-delivery systems designed to simultaneously deliver protective metabolites like SCFAs and UDCA to the tumor microenvironment represent a promising avenue for future investigation. And for prevention, interventions combining a high-fiber diet with engineered probiotics, for instance, those designed to overexpress protective metabolic pathways such as indole-3-lactic acid synthesis, hold the promise of systematically enhancing the overall protective network, thereby potentially reducing the risk of HCC [140,141]. Beyond metabolites derived from gut microbiota, the metabolic reprogramming of host cells itself constitutes a key feature of HCC and has emerged as a therapeutic target. For instance, targeting the amino acid metabolism enzyme *STAMBPL1* has been demonstrated to effectively inhibit HCC progression [159]. The net effect of a gut microbial metabolite on HCC progression thus represents a dynamic equilibrium, constantly adjusted by key contextual ‘knobs’ such as metabolite concentration, receptor expression, and the local microenvironment. This concept of a context-dependent functional balance is visualized in Figure 4.

In summary, accumulating evidence from clinical research and animal models suggests that gut microbiota-derived metabolites play a significant role in the development and progression of HCC. Furthermore, these metabolites hold promise as early diagnostic biomarkers for HCC. However, current mechanistic studies are predominantly based on animal models, and further clinical trials are warranted to confirm their translatability to humans.

## 4. Potential Roles of Gut Microbiota and Its Metabolites in HCC Diagnosis and Treatment

### 4.1. Role in HCC Diagnosis

Numerous studies have demonstrated a close association between gut microbiota dysbiosis and the occurrence and progression of HCC [53]. Specific microbial communities and their metabolites can serve as non-invasive biomarkers for early diagnosis and prognostic evaluation of HCC. Early-stage HCC patients exhibit distinct gut microbiota characteristics, including elevated α microbial diversity, elevated abundance of *Actinobacteria*, and significant changes in 13 signature genera, such as *Gemmiger* and *Parabacteroides* [55]. A diagnostic model based on 30 microbial operational taxonomic units (OTUs) achieved an area under the curve (AUC) of 0.806 in distinguishing early HCC and maintained stable performance (AUC 0.768–0.817) in cross-regional validation [55]. Metabolomic analyses identified BAs [160], such as taurochenodeoxycholic acid (TCDCA) and GCDCA, as well as SCFAs [28,55], as diagnostically valuable. Species such as *Odoribacter splanchnicus* and *Ruminococcus bicirculans* have also been identified as potential biomarkers [160]. The discovery of reliable biomarkers relies on multi-omics integration strategies. For instance, in neurodegenerative disease models, integrating fecal microbiota sequencing with multi-compartmental metabolomics enables precise localization of functional microbial-metabolite modules [161]. Applying such multi-omics approaches in HCC research will significantly enhance the accuracy of diagnostic and prognostic models. In addition to serving as an early diagnostic marker for HCC, responders to anti-PD-1 therapy exhibit higher microbial diversity and enrichment of beneficial bacteria, such as *Akkermansia* and *Ruminococcaceae*, which can predict treatment efficacy as early as 6 weeks post-therapy [162]. Multi-omics sequencing has identified specific bacterial taxa (e.g., *Actinomyces* sp. ICM47, *Senegalimassilia anaerobia*) and metabolites as prognostic markers in HCC patients undergoing immune checkpoint inhibitor (ICI) therapy [163].

These findings offer a compelling rationale for non-invasive screening, efficacy prediction, and risk stratification in HCC [28,55]. However, the clinical translation of these findings is hampered by significant challenges. Variations in metabolite detection methods and data analysis pipelines lack standardization, and most critically, single-analyte biomarkers have consistently demonstrated suboptimal performance in clinical settings. This limitation necessitates a paradigm shift towards developing composite biomarkers that reflect the microenvironmental context, for which the context-dependent framework provides a clear roadmap. The central premise is to design biomarkers that capture the functional “context” rather than isolated molecular levels. A primary strategy is to utilize metabolite ratios. For instance, the DCA/UDCA ratio may hold greater diagnostic value, as it captures the balance between the pro-tumorigenic DCA, and the hepato-protective UDCA. Similarly, the IPA-to-I3P ratio could offer profound insight into the host’s immune state, based on their opposing roles in modulating T-cell immunity [134] and direct observations of altered tryptophan metabolism in HCC patients [139]. Building on this principle, a more sophisticated approach involves creating pathway activation signatures. This involves integrating metabolite concentrations with functional readouts, such as a composite signature combining portal SCFAs levels with hepatic GPR43 mRNA expression, a receptor directly implicated in acetate-mediated tumor suppression [42] or assessing AhR activity signatures within tumor-infiltrating lymphocytes, which reflect the pro-suppressive transcriptional program driven by tumor-derived metabolites [135]. Ultimately, this approach can be expanded by combining key microbial taxa, their metabolic output, and host inflammatory markers into truly holistic, multi-domain classifiers. Looking ahead, the integration of multi-omics datasets through artificial intelligence (AI) and machine learning models will be pivotal in defining and validating these complex contextual signatures. Such context-aware biomarkers are poised to achieve higher specificity by distinguishing between disease states that may share similar metabolic alterations but differ fundamentally in their underlying functional circuitry. Realizing this vision, however, will require a concerted effort to overcome current hurdles. Future research must focus on the rigorous validation of these next-generation signatures in large-scale, multicenter clinical trials. Only then can we fully translate the insights from the microbiome and metabolome into the precision diagnosis of HCC.

### 4.2. Role in HCC Treatment

The gut microbiota and its metabolites not only hold promise for the diagnosis of HCC but also play a significant role in its treatment. During chemotherapy, they modulate host immunity and drug metabolism, thereby influencing both efficacy and toxicity [164,165]. In radiotherapy, dysbiosis can drive primary resistance by suppressing antigen presentation and the cGAS-STING-IFN-I axis, ultimately weakening effector T-cell activity [166]. Similar patterns emerge with immune-checkpoint inhibitors: antibiotic exposure reduces ICI efficacy [167,168], whereas a diverse microbiome enriched in beneficial genera (e.g., *Bifidobacterium bifidum*) correlates with superior progression-free and overall survival [169]. In contrast, over-representation of *Bacteroides intestinalis* compromises therapeutic benefit [170,171]. Emerging predictive models that integrate community dynamics [162] and metabolite signatures [172] may refine real-time treatment monitoring.

Engineered microbial communities offer a novel therapeutic avenue for HCC by designing strains that secrete anticancer metabolites or directly modulate host physiology. Modified bacteria such as *Escherichia coli*, *Lactobacillus*, and *Salmonella* can deliver short-chain fatty acids, indole derivatives, or pathway-specific inhibitors. Engineered Salmonella that homes in on tumors and releases an IL-15/FlaB fusion protein exemplifies this concept, boosting antitumor immunity via TLR4/5 activation [173,174]. Focused-ultrasound gating provides spatial and temporal control of bacterial activity, limiting collateral damage while amplifying immune activation [175]. In parallel, genetically and chemically engineered probiotics reshape microbiota and have already shown benefit in inflammatory bowel disease, supporting their exploration in HCC [176]. Key challenges, strain safety, off-target effects, and scalable manufacture, must be addressed. Future work should include rigorously designed clinical trials, advanced delivery vehicles (e.g., micro-encapsulation), and combination regimens with current standards such as sorafenib or lenvatinib.

Beyond engineered bacteria, conventional probiotics (*Bifidobacterium*, *Lactobacillus*) and prebiotics can fortify the intestinal barrier, curb bacterial translocation, and dampen hepatic inflammation and fibrosis. Trials with *Bifidobacterium longum* report faster recovery, shorter hospitalization, and improved 1-year survival [44]. Similar benefits in NAFLD support their adjunctive use in HCC [177,178,179,180]. Cutting-edge approaches, such as nano-carriers that deliver microbial metabolites (SCFAs, BAs), further boost antitumor immunity and constrain tumor growth [181]. *B. longum* appears to mediate these effects via serotonin, secondary bile acids, and SCFAs pathways [44]. These microbiota-based strategies could complement kinase inhibitors already in use (sorafenib, lenvatinib) and those under investigation for MAPK and JAK-STAT blockade. Since targeted drugs may not be effective for patients over the long term and may lead to drug resistance, it is important to identify biomarkers that predict treatment response and develop new treatment strategies.

Fecal microbiota transplantation (FMT) is a procedure that restores gut microbial balance by transferring microbiota from healthy donors to patients [182]. In the context of HCC, FMT enhances immune responses by modulating bile acid metabolism and influencing immune cell function [30]. Studies have demonstrated that FMT can increase the abundance of beneficial bacteria, such as *Anaerotruncus colihominis* and *Dysosmobacter welbionis*, improve intestinal dysbiosis, and subsequently inhibit neutrophil inflammatory activation, excessive NET formation, tumor vascular growth, and tissue necrosis in liver metastatic tumors. This effectively slows disease progression in HCC mouse models [65]. Clinical trials indicate that FMT combined with anti-PD-1 therapy demonstrates efficacy in some solid tumor patients resistant to PD-1 inhibitors (overall disease control rate of 46.2%). The potential benefits for HCC patients require further validation to achieve disease control, highlighting the therapy’s potential in overcoming resistance to immune checkpoint inhibitors [183,184]. However, the application of FMT in HCC treatment still faces several challenges. Currently, existing randomized trials are limited in scale, there are no standardized criteria for donor selection. The substantial heterogeneity in the efficacy of FMT precisely corroborates the principle of context-dependency [185]. A successful FMT likely does not hinge on the engraftment of a single ‘super-bug,’ but rather on the establishment of a new ecosystem capable of producing a beneficial metabolic profile that is compatible with the patient’s individual context.

Beyond these therapeutic avenues, the context-dependent framework presented herein offers a vital paradigm shift for precision medicine in HCC. A prime example is the targeted application of FXR agonists, such as obeticholic acid, for the specific prevention of NASH-associated HCC [186]. Crucially, this therapy’s efficacy is contingent on the patient’s specific ‘receptor expression environment,’ rather than universal applicability. The pathological progression from NASH to HCC is fundamentally driven by impaired steady-state FXR signaling, a defect exacerbated by altered bile acid profiles and gut dysbiosis [187]. By administering potent synthetic FXR agonists, this intervention directly addresses this core vulnerability, compensating for endogenous ligand deficiency and reactivating the homeostatic FXR pathway [186]. This precision targeting of a specific mechanistic deficit within the NASH context predicts multifaceted benefits: restoring negative feedback on bile acid synthesis (thereby limiting toxic secondary BA production), suppressing hepatic inflammation, and ameliorating liver fibrosis, all critical factors in mitigating HCC risk. Importantly, this strategy underscores that therapeutic success can be achieved not by directly targeting cancer cells, but by rectifying the underlying metabolic and signaling dysregulations within the host microenvironment that foster tumorigenesis [102]. This context-specific approach highlights its potential limitations in HCC of different etiologies, such as viral hepatitis, where FXR signaling defects may not be the primary pathogenic driver.

Collectively, the gut microbiota and its metabolites significantly influence all facets of HCC management, from diagnosis to prognosis and therapy. Biomarker discoveries and interventions such as probiotics, engineered microbes, metabolite delivery, and FMT are poised to transform clinical practice. Future advancements will necessitate mechanistic dissection, protocol optimization, and patient-specific strategies to translate these insights into tangible clinical benefits.

## 5. Discussion and Future Perspectives

This review integrates bile acids, short-chain fatty acids, trimethylamine N-oxide, indole metabolites, and lipopolysaccharide within a unified “context-dependency” framework. This study systematically elucidates how a single molecule undergoes bidirectional switching between protective and tumor-promoting functions under the synergistic regulation of four control “knobs”, concentration-time window, receptor expression profile, bacterial strain, and microenvironmental pH/oxygen status, and why different molecules within the same family exhibit distinct functionalities. This framework reconciles several contradictory observations in the clinical literature and provides a molecular-level mechanism for phenomena such as why a high-fiber diet is effective in non-alcoholic fatty liver disease-associated HCC but not in hepatitis B virus-related HCC.

**Figure 5:**
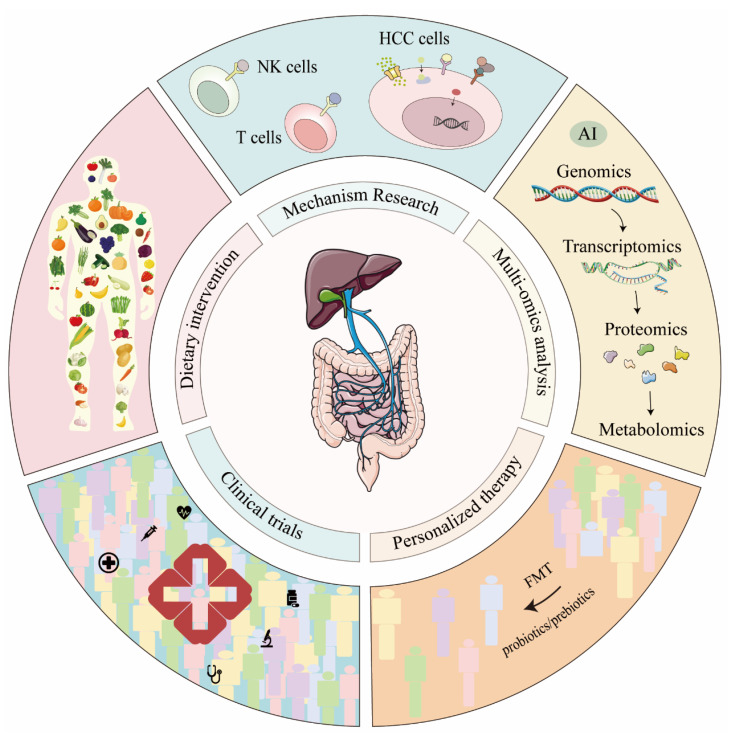
Based on the current research progress on gut microbiota and liver cancer, future research directions are outlined. Targeting specific metabolic or immune pathways to identify new drug targets may provide novel strategies for HCC treatment. Integrating multi-omics approaches, such as genomics, proteomics, and metabolomics, will help comprehensively understand disease mechanisms. Large-scale clinical trials are crucial for validating the safety and efficacy of microbiota interventions. Additionally, personalized microbiota treatment plans, including optimizing the use of probiotics, prebiotics, and FMT, as well as exploring the therapeutic potential of microbial metabolites, are promising areas for future research. The role of dietary interventions, such as high-fiber diets, in the prevention and treatment of HCC should also be further investigated. In summary, translating microbiota and HCC research into practical diagnostic and therapeutic strategies will require multifaceted efforts and exploration.

The “context-dependency” framework proposed in this review not only unifies findings from basic research but also has important implications for clinical practice, suggesting the need for a paradigm shift from single-target thinking to a systems regulation strategy. This is particularly evident in the development of diagnostic biomarkers. The efficacy of relying on a single metabolite, such as TMAO, is generally suboptimal [60]. However, when the perspective shifts to a multi-dimensional metabolite fingerprint that reflects the overall “metabolic context,” diagnostic accuracy may be substantially improved [42,139]. For example, we can focus on the ratio of DCA to UDCA and the ratio of IPA to I3P. The same logic applies to therapeutic interventions. A seemingly straightforward intervention like antibiotic use, while potentially solving one problem in the short term (e.g., reducing LPS), can paradoxically lead to long-term negative clinical consequences because it disregards the disruption of the entire protective metabolic network, such as the butyrate-indole axis, as evidenced in preclinical models [115]. Conversely, proactively and precisely modulating the “context” can improve efficacy for existing therapies like immune checkpoint inhibitors. In immunologically “cold” tumors, reshaping the tumor microenvironment and enhancing T cell stemness with microbial metabolites like IPA can transform an otherwise ineffective immunotherapy into a highly effective anti-tumor response. Collectively, these examples demonstrate that future HCC management strategies must move beyond simplistic dichotomies of good and bad and pivot towards the precise assessment and dynamic intervention of the patient’s individual context [134]. Figure 5 emphasizing the microbiota-gut–liver axis as a unified framework for next-generation HCC diagnosis and treatment.

Looking ahead, several critical questions arise. How can we leverage cutting-edge technologies like single-cell sequencing combined with spatial metabolomics to characterize the key “microenvironmental context” that dictates metabolite function at single-cell resolution? Can we develop AI-driven multi-omics models that integrate microbiome, metabolome, and clinical data to predict the specific “contextual state” of a patient’s gut–liver axis, thereby enabling precision stratification? And can we design “context-responsive” engineered probiotics, for instance, a “smart bacterium” that commences secretion of anti-tumor metabolites, such as butyrate, only upon detecting specific HCC microenvironmental signals like particular inflammatory cytokines?

## Figures and Tables

**Table 1:** Changes in microbiota composition associated with HCC in animal models.

Model	Disease	Increase	Decrease	Source	Reference
mice	NAFLD-HCC	*Mucispirillum*, *Desulfovibrio*, *Anaerotruncus*	*Bifidobacterium*, *Bacteroides*	feces	[39]
mice	Liver cancer		*Bifidobacterium*, *Bacteroides*	feces	[40]
mice	Cancer cachexia	*Enterobacteriaceae*	*Lachnospiraceae*	feces	[41]
mice	NAFLD-HCC		*B. pseudolongum*	feces	[42]
mice	HCC		*Lactobacillus reuteri*	feces	[43]
mice	HCC		*Akkermansia muciniphila*	feces	[32]
mice	HCC		*Bifidobacterium longum*	feces	[44]
mice	Obesity induced HCC	dysbiosis		Feces and intestinal contents	[45]
mice	NASH-HCC	*Atopobium* spp., *Bacteroides* spp., *Bacteroides vulgatus*, *B. acidifaciens*, *B. uniformis*, *Clostridium cocleatum*, *C. xylanolyticum*, *Desulfovibrio* spp.	*Bifidobacterium longum*	feces	[46]
rat	DEN induced HCC	*Enterobacteriaceae*	*Bifidobacterium* spp.	feces	[47]
rabbit	VX2 HCC	*Bacteroidaceae*, *Prevotellaceae*, *Flavobacteriaceae*, *Flavobacteriales*, *Alistipes, Marseille*	*Ruminiclostridium*, *Christensenellaceae*, *Enterorhabdus*, *Christensenellaceae*, *Mucispirillumgenera*	feces	[48]
mice	HCC	dysbiosis		feces and intestinal contents	[49]
mice	MYC transgenic spontaneous HCC	*Gram-positive bacteria*, *Bacteria mediating primary-to-secondary bile acid conversion*, *Clostridium scindens*		feces	[50]
mice	HCC	Gram-positive bacteria		feces	[51]

**Table 2:** Changes in microbiota composition associated with HCC in human studies.

Model	Disease	Increase	Decrease	Source	Reference
human	HCC	*Bacteroides*, *Ruminococcaceae*	*Bifidobacterium*	feces	[52]
human	NBNC-HCC	*Bacteroides*, *Streptococcus*, *Ruminococcus gnavus group*, *Veillonella*, *Erysipelatoclostridium*	*Romboutsia*, *UCG-002*, *Lachnospiraceae NK4A-136*, *Eubacterium hallii group*, *Lachnospiraceae ND-3007 group*, *Erysipelotrichaceae UCG-003*, *Bilophila*	feces	[53]
human	HCC		*Bifidobacterium longum*	feces	[44]
human	HCC and ICC		*Ruminococcaceae*, *Porphyromonadaceae*, *Bacteroidetes*	feces	[54]
human	HCC	*Klebsiella*, *Haemophilus*, *Clostridium sensu stricto*, *Megasphaera*, *Acidaminococcus*, *Lactobacillus*	*Alistipes*, *Phascolarctobacterium*, *Ruminococcus*, *Oscillibacter*, *Coprococcus*, *Bilophila*, *ClostridiumIV*, *Butyricicoccus*, *Anaerostipes*, *Akkermansia*, *Allisonella*, *Coprobacillus*	feces	[55]
human	HCC	*Lachnoclostridium*		feces	[56]
human	HCC	*Escherichia coli*		feces	[57]
human	HCC	*Proteobacteria*, *Desulfococcus*, *Enterobacter*, *Prevotella*, *Veillonella*	*Cetobacterium*	feces	[58]
human	PLC	*Enterobacter ludwigii*, *Enterococcaceae*, *Lactobacillales*, *Bacilli*, *Gammaproteobacteria*, *Veillonella*	*firmicutes*, *bacteroidetes*, *Clostridia*	feces	[59]
human	PLC	dysbiosis		feces	[60]
human	HCC	*Neisseria*, *Enterobacteriaceae*, *Veillonella*, *Limnobacter*	*Enterococcus*, *Phyllobacterium*, *Clostridium*, *Ruminococcus*, *Coprococcus*	feces	[61]
human	HCC	*Proteobacteria*, *Enterobacteriaceae*, *B. xylanisolvens*, *B. caecimuris*, *Ruminococcus gnavus*, *Clostridium bolteae*, *Veillonella parvula*	*Oscillospiraceae*, *Erysipelotrichacea*	feces	[62]

## Data Availability

No new data were created or analyzed in this study. Data sharing is not applicable to this article.
